# Efficacy and safety of nanoparticle albumin‐bound paclitaxel monotherapy after immune checkpoint inhibitor administration for advanced non‐small cell lung cancer: A multicenter Phase 2 clinical trial

**DOI:** 10.1002/cam4.5978

**Published:** 2023-04-20

**Authors:** Tomoaki Sonoda, Yukihiro Umeda, Yoshiki Demura, Toshihiko Tada, Koki Nakashima, Masaki Anzai, Makiko Yamaguchi, Akikazu Shimada, Masahiro Ohi, Chisato Honjo, Yuko Waseda, Masaya Akai, Tamotsu Ishizuka

**Affiliations:** ^1^ Third Department of Internal Medicine, Faculty of Medical Sciences University of Fukui Fukui Japan; ^2^ Department of Respiratory Medicine Japanese Red Cross Fukui Hospital Fukui Japan; ^3^ Department of Respiratory Medicine Municipal Tsuruga Hospital Fukui Japan

**Keywords:** immune checkpoint inhibitor, nab‐paclitaxel, non‐small cell lung cancer, programmed cell death 1 inhibitor, programmed death ligand 1 inhibitor

## Abstract

**Background:**

Whether immunotherapy improves the efficacy or worsens adverse events of subsequent chemotherapy remains unclear. We performed a Phase 2 study to evaluate the efficacy and safety of nanoparticle albumin‐bound paclitaxel (nab‐paclitaxel) as a treatment for advanced non‐small cell lung cancer (NSCLC) after treatment with programmed cell death 1 or programmed death ligand 1 [PD‐(L)1] inhibitor failure.

**Methods:**

Nab‐paclitaxel (100 mg/m^2^) was administered on Days 1, 8, and 15 of a 28‐day cycle to patients with advanced NSCLC within 12 weeks after the failure of PD‐(L)1 inhibitor treatment. The primary endpoint was objective response rate (ORR) in all patients; the secondary endpoints were disease control rate (DCR), progression‐free survival (PFS), overall survival (OS), and safety.

**Results:**

Thirty cases were registered, and 29 cases were included in the analysis. The ORR was 55.2% (95% confidence interval [CI]: 28.1%–79.6%) and the DCR was 86.2% (95% CI: 65.9%–97.0%). The median PFS was 5.6 months (95% CI: 4.4–6.7 months), and PFS rates at 1‐ and 2‐year timepoints were 34.5% and 13.3%, respectively. The median OS was 11.9 months (95% CI: 0.8–23.0 months). Good performance status and responder of previous PD‐(L)1 inhibitor therapy were independent predictors of PFS. Grade 3 or higher toxicities included leukopenia (27.6%), neutropenia (31.0%), peripheral sensory neuropathy (6.9%), increased alanine aminotransferase and aspartate aminotransferase levels (3.4%), and interstitial lung disease (3.4%).

**Conclusions:**

Nab‐paclitaxel therapy improved ORR after PD‐(L)1 inhibitor treatment failure with a durable response of 13% and acceptable toxicities in patients with advanced NSCLC.

## 1 INTRODUCTION

Globally, lung cancer was the leading cause of cancer‐related mortality in 2020, contributing to approximately 1.8 million deaths.^1^ In recent years, amidst the ongoing development of various drugs, immunotherapy has emerged as a novel treatment option for advanced non‐small cell lung cancer (NSCLC), either alone or in combination with cytotoxic chemotherapy. Furthermore, it has resulted in improved prognosis for patients with advanced NSCLC.^2^ However, the therapeutic effects after second‐line treatment remain unsatisfactory,^3^ and further treatment strategies are urgently required.

Notably, the results of clinical trials of immune checkpoint inhibitors (ICIs), including programmed cell death 1 (PD‐1) and programmed death ligand 1 (PD‐L1) inhibitors, indicate that they are expected to significantly improve overall survival (OS) despite a lower objective response rate (ORR).^4^, ^5^, ^6^ These reproducible results suggest that ICIs have not only direct antitumor effects, but also a positive post‐treatment impact. In fact, several retrospective studies have reported a possible enhancement in the effect of chemotherapy used immediately after ICI administration,^7^, ^8^, ^9^, ^10^, ^11^ whereas another study has suggested no enhancement.^12^ All of these reports are retrospective studies and include various anticancer agents, leaving the matter inconclusive.

Commonly used anticancer agents for previously treated patients with advanced NSCLC include docetaxel, pemetrexed, gemcitabine, and a combination of docetaxel and ramucirumab. However, nanoparticle albumin‐bound paclitaxel (nab‐paclitaxel) has recently demonstrated significantly longer progression‐free survival (PFS) than docetaxel^13^ and is considered a standard second‐line treatment. Furthermore, the hazard ratio (HR) for OS of docetaxel plus ramucirumab therapy, currently considered the most potent second‐line therapy, versus docetaxel alone was 0.86,^14^ and the HR for nab‐paclitaxel versus docetaxel was 0.85.^13^ This suggests that nab‐paclitaxel therapy is one of the most effective existing second‐line therapies. In terms of adverse events, nab‐paclitaxel may be safer than docetaxel plus ramucirumab because febrile neutropenia occurs less frequently with nab‐paclitaxel than with docetaxel plus ramucirumab (2% vs. 16%).^13^, ^14^ These considerations suggest that nab‐paclitaxel monotherapy may be more suitable as a treatment for previously treated patients with advanced NSCLC.

Several chemotherapy and PD‐1 or PD‐L1 [PD‐(L)1] inhibitor combination regimens are currently available for patients with advanced NSCLC. Among these, the combination of nab‐paclitaxel and carboplatin with a PD‐(L)1 inhibitor has been reported to be both effective and well tolerated.^15^, ^16^ This suggests that nab‐paclitaxel may not increase the probability of adverse events when administered after ICI treatment. Therefore, the purpose of this study was to prospectively evaluate the efficacy and safety of nab‐paclitaxel monotherapy immediately after PD‐(L)1 inhibitor treatment failure in a Phase 2 study of patients with advanced NSCLC.

## 2 MATERIALS AND METHODS

### 2.1 Patients

The criteria for patient eligibility included an age of 20 years or older, diagnosis of advanced or recurrent NSCLC confirmed histologically or cytologically, within 12 weeks after PD‐(L)1 inhibitor (single or combination therapy) treatment failure due to disease progression or unacceptable adverse events, an Eastern Cooperative Oncology Group performance status (ECOG‐PS) of 0–2, and measurable lesions according to Response Evaluation Criteria in Solid Tumor (RECIST) version 1.1.^17^ Furthermore, the patients should have had no severe disorders in major organs (bone marrow, heart, lungs, liver, and kidneys). The baseline criteria for enrollment by organ function were as follows: a white blood cell count ≥3000/mm^3^, neutrophil count ≥1500/mm^3^, hemoglobin content ≥9.0 g/dL, platelet count ≥100,000/mm^3^, aspartate aminotransferase (AST) and alanine aminotransferase (ALT) levels ≤100 IU/L, total bilirubin concentration ≤1.5 mg/dL, creatinine concentration ≤1.5 mg/dL, urine protein level ≤1+, prothrombin time‐international normalized ratio ≤1.5, and resting arterial oxygen saturation in room air ≥90%. A life expectancy of more than 3 months was required. Previous treatment with molecular‐targeted drugs for driver oncogenes, radiotherapy, and chemotherapy without solvent‐based paclitaxel or nab‐paclitaxel was permitted.

The key exclusion criteria included patients who had a history of severe drug allergies or hypersensitivity to nab‐paclitaxel or albumin; had been previously treated with solvent‐based paclitaxel or nab‐paclitaxel; were receiving more than 10 mg of systemic prednisolone or an equivalent amount of another corticosteroid at the time of registration; had Grade 2 or higher peripheral neuropathy; had interstitial pneumonia (apparent on chest X‐ray); had pleural effusions, ascites, and/or pericardial effusions requiring drainage; had symptomatic central nerve metastasis; received therapeutic or palliative radiation therapy within 2 weeks of study Day 1; underwent major surgery within 4 weeks of study Day 1; had a concurrent active malignancy; were lactating/breastfeeding; had a positive pregnancy test; and/or were positive for hepatitis B surface antigen(s).

### 2.2 Study design and treatment

This trial was a three‐center, open‐label, single‐group, Phase 2 study. After confirming the baseline assessment, patients received a 30‐min infusion of nab‐paclitaxel (Abraxane®, Taiho Pharmaceutical Co., Ltd.) at a dose of 100 mg/m^2^ on Days 1, 8, and 15 (every 4 weeks). At the beginning of the second and subsequent cycles, patients had to meet the following criteria: a neutrophil count ≥1500/mm^3^, platelet count ≥10,000/mm^3^, hemoglobin ≥9.0 g/dL, AST and ALT ≤2.5 times the upper limit of normal, total bilirubin concentration ≤1.5 mg/dL, creatinine concentration ≤1.5 mg/dL, and peripheral neuropathy of ≤Grade 2. Prior to administration of nab‐paclitaxel on Days 8 and 15, patients had to meet the following criteria: a neutrophil count ≥500/mm^3^, platelet count ≥50,000/mm^3^, and peripheral neuropathy of ≤Grade 2. Dose reductions of 20 mg/m^2^ (by a minimum of 60 mg/m^2^) were allowed if the following criteria were met during treatment: Grade 4 neutropenia, Grade 3 or higher neutropenia that delayed the start of each cycle by at least 7 days, Grade 3 or higher thrombocytopenia, febrile neutropenia, Grade 2 or higher skin disorders, and/or Grade 3 or higher nonhematologic toxicities other than alopecia. Treatment continued until progressive disease (PD) was confirmed or unacceptable adverse events were identified.

### 2.3 Study oversight

This study was approved by the Research Ethics Committee of the University of Fukui (No. 20170185), the Institutional Review Board of the Japanese Red Cross Fukui Hospital, and the Institutional Review Board of the Municipal Tsuruga Hospital (No. 248). It was performed in accordance with the Declaration of Helsinki and the guidelines for Good Clinical Practice. All patients provided written informed consent. The clinical trial was registered at https://www.umin.ac.jp/ctr/index.htm (UMIN000030994). This trial was self‐funded and designed by the principal investigators. All authors vouch for the accuracy and completeness of the data and for the adherence of the experiments performed for this research paper to the described protocol.

### 2.4 Endpoints and assessments

Disease assessments using RECIST version 1.1^17^ were performed using computed tomography or magnetic resonance imaging (MRI) scans of the chest and abdomen at intervals of 8 weeks or less until PD. The response was confirmed at least 4 weeks after the initial response. Although brain MRI scanning was performed in all patients at the time of NSCLC diagnosis, it was not mandated at the time of inclusion except in patients with known or suspected brain metastases. In patients with brain metastases, a brain MRI was also performed at intervals of 8 weeks or less during nab‐paclitaxel treatment.

The primary endpoint was ORR as determined via investigator assessments according to RECIST version 1.1.^17^ Response assessments via imaging were mutually confirmed by the principal investigators at each site.

The secondary endpoints were disease control rate (DCR), PFS, which is the time from the date of enrollment to the date of disease progression, OS, which is the time from the date of enrollment to the date of death from any cause, and safety. Adverse events were evaluated in accordance with the National Cancer Institute Common Terminology Criteria for Adverse Events Version 4.0.

In subset analyses, we examined ORR, PFS, and OS by age, sex, ECOG performance status, histological type (nonsquamous or squamous cell carcinoma), history of smoking (current smoking, past smoking or non‐smoking), the degree of PD‐L1 expression, and number of previous regimens.

### 2.5 Statistical analysis

Response was summarized with the use of frequency counts and percentages, with exact 95% confidence intervals (CIs) calculated. Fisher's exact test was used to examine the association between two categorical variables. PFS and OS were estimated using the Kaplan–Meier method, and 95% CIs were calculated. The Cox proportional hazards model using the stepwise method was used to identify prognostic factors. Statistical analysis was performed using SPSS Statistics V.26.0 (IBM). *p* < 0.05 indicated significant differences.

To calculate the sample size, one arm binomial was used in SWOG's Statistical Tools (https://stattools.crab.org/index.html). At the time of study design, nab‐paclitaxel therapy was not established as a standard second‐line therapy; thus, docetaxel was used as the reference. Shepherd et al. reported an ORR of 7.1% for docetaxel second‐line therapy,^18^ and in our previous study, we found that nab‐paclitaxel alone as a second‐line treatment had an ORR of 28.1%.^19^ Because the present study was designed for second and third line and beyond, it is assumed that the threshold value is 0.07 and the expected value is 0.25. When the required number of subjects was calculated with *α* = 0.05 (one side) and *β* = 0.2, the required number of subjects was 24. Therefore, considering some ineligible cases, the planned number of cases was set to 30.

## 3 RESULTS

### 3.1 Patient characteristics

**FIGURE 1:**
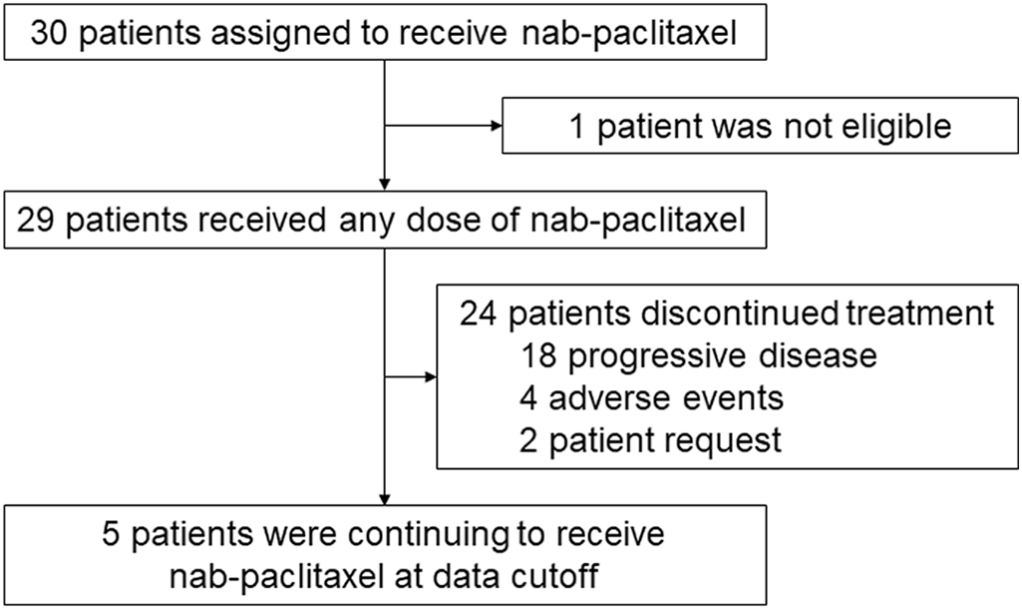
Flow chart of patient characteristics.

Between February 2018 and December 2020, 30 patients with advanced NSCLC were recruited, and 29 patients were included in the analysis (Figure 1). The protocol‐defined final analysis was performed on December 11, 2021. Table 1 shows the baseline demographic and clinical characteristics of the patients. Seventy percent subjects were male, and the ages of the subjects ranged from 43 to 82 years. ECOG‐PS, histology, and PD‐L1 status were as follows: 9 patients with PS 0, 19 patients with PS 1, 1 patient with PS 2; 11 patients with squamous cell carcinoma, 14 patients with adenocarcinoma, 4 patients with NSCLC, not otherwise specified; 13 patients with PD‐L1 <1%, 5 patients with PD‐L1 1%–49%, 9 patients with PD‐L1 ≥50%, and 2 patients with unknown PD‐L1 status. Past treatment history was 1 regimen in 6 patients, 2 regimens in 17 patients, and 3 or more regimens in 6 patients. Postoperative adjuvant chemotherapy was not counted as a past treatment line. Epidermal growth factor receptor (*EGFR*) mutations were positive for the driver oncogene in only three patients. Twenty‐eight patients terminated ICI treatment due to confirmed PD and subsequently received nab‐paclitaxel therapy. In only one patient, nivolumab was discontinued due to Grade 3 liver enzyme increase; thus, nab‐paclitaxel was administered before PD was confirmed. In this case, the liver injury improved spontaneously and no steroids were administered.

**TABLE 1:** Patient characteristics.

Characteristics	*N* (%)
Age (years), median (range)	69 (43–82)
Sex	
Male	22 (75.9)
Female	7 (24.1)
ECOG performance status	
0	9 (31.0)
1	19 (65.5)
2	1 (3.4)
Clinical stage	
III	8 (27.6)
IV	18 (62.1)
Recurrent	3 (10.3)
Smoking status	
Never	4 (13.8)
Past	20 (69.0)
Current	5 (17.2)
Histology	
Squamous cell carcinoma	11 (37.9)
Adenocarcinoma	14 (48.3)
NOS	4 (13.8)
*EGFR* mutation status	
Positive	3 (10.3)
Negative	26 (89.7)
PD‐L1 status	
<1%	13 (44.8)
1%–49%	5 (17.2)
≥50%	9 (31.0)
Unknown	2 (6.9)
Number of previous regimens	
1	6 (20.7)
2	17 (58.6)
≥3	6 (20.7)
Type of pervious systemic anticancer therapy	
Carboplatin+pemetrexed+pembrolizumab	2 (6.9)
PD‐1 antibody	23 (79.3)
PD‐L1 antibody	4 (13.8)
Confirmed response of previous ICI treatment	
Partial response	10 (34.5)
Stable disease	7 (24.1)
Progressive disease	12 (41.4)
Reasons for termination of ICI treatment	
Confirmed progressive disease	28 (96.6)
Adverse events	1 (3.4)

Abbreviations: ECOG, Eastern Cooperative Oncology Group; *EGFR*, epidermal growth factor receptor; ICI, immune‐checkpoint inhibitor; NOS, not otherwise specified; PD‐1, programmed death 1; PD‐L1, programmed death ligand 1.

### 3.2 Efficacy

**FIGURE 2:**
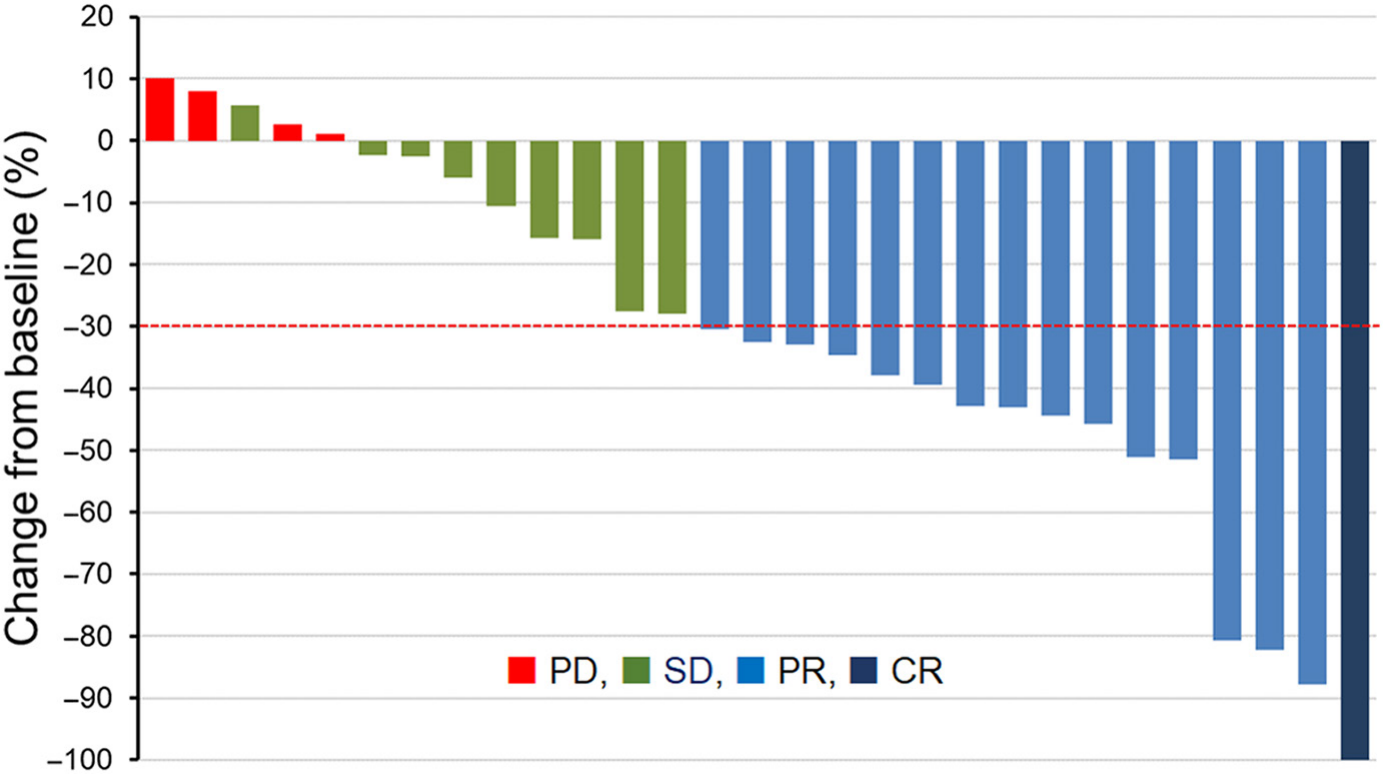
Waterfall plots for the best percentage change in target lesion size from the baseline are shown for all patients. The patterns of each bar indicate the best overall response assessed by the response evaluation criteria in solid tumors version 1.1.

**FIGURE 3:**
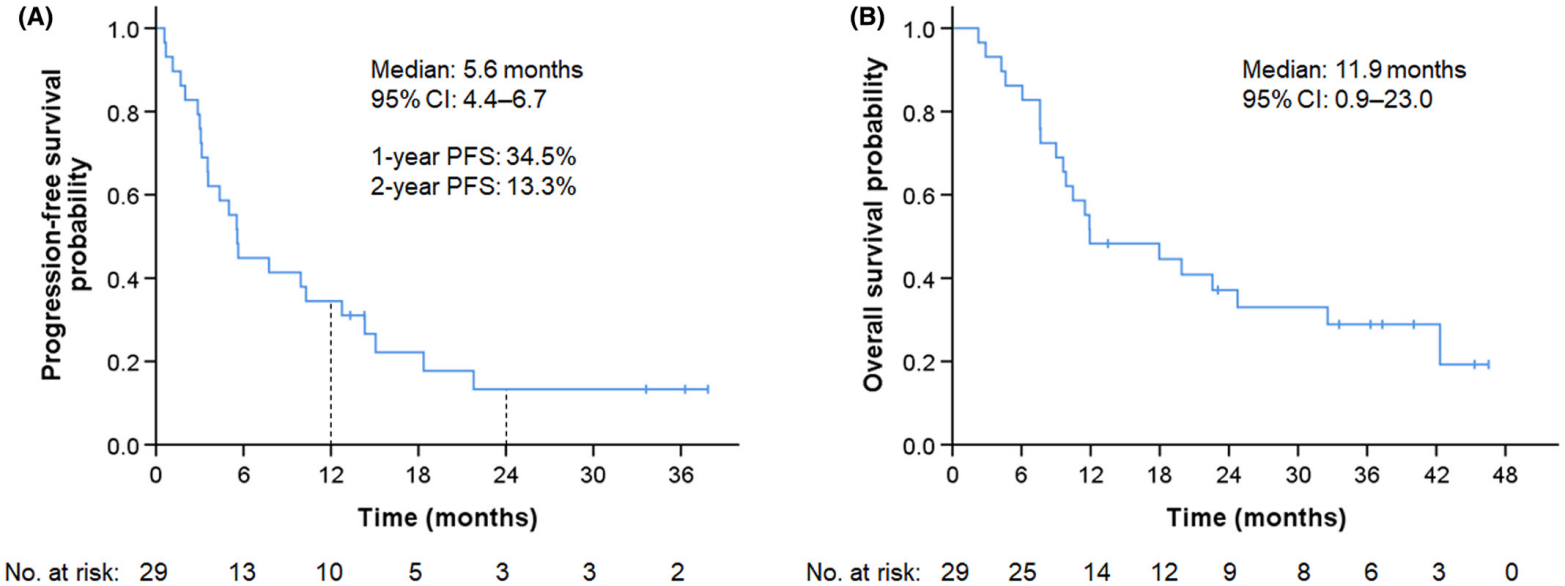
Kaplan–Meier estimates for progression‐free survival (A) and overall survival (B) of all patients.

Of the 29 assessable patients, 1 (3.4%) achieved complete response (CR), 15 (51.7%) had partial response (PR), 9 (31.0%) had stable disease (SD), and 4 (13.8%) experienced PD. The ORR was 55.2% (95% CI: 28.1%–79.6%) and the DCR was 86.2% (95% CI: 65.9%–97.0%), suggesting high efficacy (Table 2). Waterfall plots for best percentage changes in target legions are shown in Figure 2. Figure 3 shows the Kaplan–Meier curves for PFS and OS. With a median follow‐up time of 12.0 months (range: 2.3–46.5 months), 24 patients showed disease progression, and 21 died from lung cancer. The median PFS was 5.6 months (95% CI: 4.4–6.7 months). PFS rates at 1 and 2 years were 34.5% and 13.3%, respectively. The median OS was 11.9 months (95% CI: 0.8–23.0 months).

**TABLE 2:** Treatment outcomes.

	No. of patients (*N* = 29)
Objective response rate, *n* (%)	16 (55.2)
[95% CI]	[28.1–79.6]
Best overall response, *n* (%)	
Complete response	1 (3.4)
Partial response	15 (51.7)
Stable disease	9 (31.0)
Progressive disease	4 (13.8)
Disease control rate, *n* (%)	25 (86.2)
[95% CI]	[65.9–97.0]
1‐year PFS rate, %	34.5
2‐year PFS rate, %	13.3
Median treatment cycles (range)	6 (1–38)

Abbreviations: CI, confidence interval; ECOG, Eastern Cooperative Oncology Group; PD‐L1, programmed death ligand 1; PFS, progression‐free survival.

**FIGURE 4:**
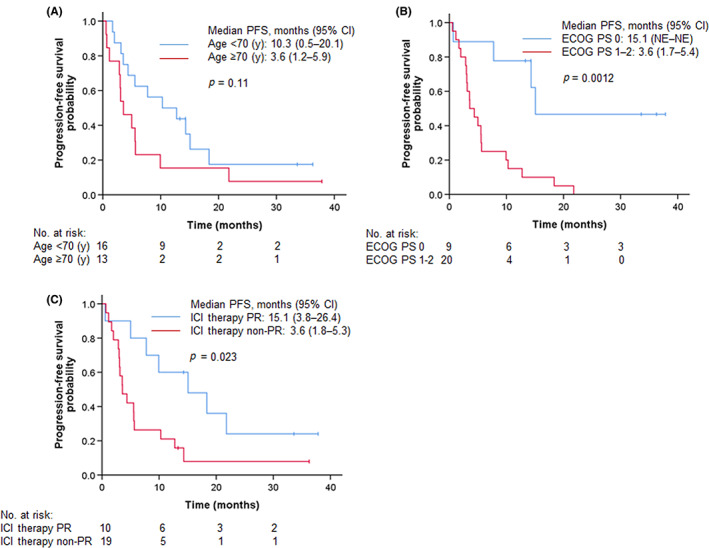
Kaplan–Meier estimates for progression‐free survival stratified by age (A), ECOG‐PS (B), and confirmed response to previous ICI therapy (C).

**FIGURE 5:**
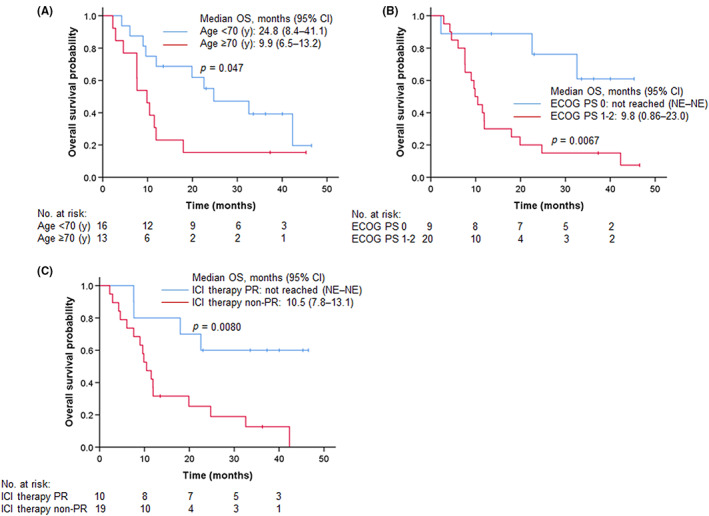
Kaplan–Meier estimates for overall survival stratified by age (A), ECOG‐PS (B), and confirmed response to previous ICI therapy (C).

Although no statistically significant differences were observed, younger patients (<70 years) and those with PS 0, squamous cell carcinoma, and 1 previous regimen tended to have a good response, while patient populations with apparently low ORR (<40%) could not be identified (Table 3). Interestingly, PD‐L1 expression did not affect the response to nab‐paclitaxel. A univariate Cox proportional hazard model revealed that ECOG‐PS 0 (HR 0.19, 95% CI 0.06–0.57, *p* = 0.003) and responder of previous ICI treatment (HR 0.36, 95% CI 0.14–0.90, *p* = 0.028) were indicators of longer PFS (Table 4). Multivariate analysis showed that ECOG‐PS 0 and responder of previous ICI treatment were independent predictors of PFS. Similarly, univariate analysis showed that ECOG‐PS 0 (HR 0.21, 95% CI 0.06–0.72, *p* = 0.013) and responder of previous ICI treatment (HR 0.25, 95% CI 0.08–0.75, *p* = 0.014) were indicators of longer OS, and multivariate analysis showed age (<70 years) and responder of previous ICI treatment as independent predictors of OS. In contrast, no significant associations were observed between PD‐L1 expression and both PFS and OS. Figure 4 shows the Kaplan–Meier curves for PFS stratified by each item in the subset analysis. Patients with PS 0 (15.1 vs. 3.6 months, *p* = 0.0012) and responder of previous ICI treatment (15.1 vs. 3.6 months, *p* = 0.023) had significant longer median PFS. Similarly, longer median OS was observed in patients with younger age (24.8 vs. 9.9 months, *p* = 0.047), PS 0 (not reached vs. 9.8 months, *p* = 0.0067), and responder of previous ICI treatment (not reached vs 10.5 months, *p* = 0.0080) (Figure 5). In addition, three cases of *EGFR*‐mutated lung cancer were included in this study, and all three cases showed response (1 case of CR, 2 cases of PR). Three patients (10.3%) received taxane‐based anticancer agents as a prior therapy in combination with docetaxel and ramucirumab, and all three patients showed a response PR to nab‐paclitaxel therapy.

**TABLE 3:** Treatment outcomes.

Variables	No. of patients who responded, *N* (%)	*p*
Age, years		
<70	11/17 (64.7)	0.31
≥70	5/12 (41.7)	
ECOG performance status		
0	6/9 (66.7)	0.34
1–2	10/20 (50.0)	
Histology		
Squamous cell carcinoma	7/11 (63.6)	0.37
Non‐squamous cell carcinoma	9/18 (50.0)	
Smoking status		
Never	2/4 (50.0)	0.62
Ever	14/25 (56.0)	
PD‐L1 status		
<1%	7/13 (53.8)	0.57
≥1%	7/14 (50.0)	
No. of previous regimens		
1	4/6 (66.7)	0.44
≥2	12/23 (52.2)	
Confirmed response to previous ICI treatment		
PR	6/10 (60.0)	0.51
SD or PD	10/19 (52.6)	

Abbreviations: ECOG, Eastern Cooperative Oncology Group; ICI, immune‐checkpoint inhibitor; PD, progressive disease; PD‐L1, programmed death ligand 1; PR, partial response; SD, stable disease.

**TABLE 4:** Prognostic factors of nab‐paclitaxel therapy using Cox models.

		PFS							OS					
		Univariate analysis			Multivariate analysis				Univariate analysis			Multivariate analysis		
	Median PFS (months)	HR	95% CI	*p*	HR	95% CI	*p*	Median OS (months)	HR	95% CI	*p*	HR	95% CI	*p*
Age (years), <70/≥70	10.3/3.6	0.52	0.23–1.17	0.11				24.8/9.9	0.42	0.18–1.01	0.054	0.31	0.11–0.89	0.029
ECOG‐PS, 0/1–2	15.1/3.6	0.19	0.06–0.57	0.003	0.19	0.06–0.58	0.004	NR/9.8	0.21	0.06–0.72	0.013	0.21	0.10–1.42	0.15
Smoking status, never/ever	3.5/5.6	1.22	0.36–4.19	0.75				10.5/18.0	1.42	0.41–4.93	0.58			
Histology, sq/non‐sq	7.8/4.4	0.67	0.29–1.54	0.34				18.0/10.5	0.67	0.28–1.58	0.36			
Tumor PD‐L1 expression (%), ≥1/<1	9.9/3.5	0.64	0.27–1.48	0.30				18.0/9.6	0.68	0.27–1.68	0.40			
Number of previous regimens, 1/≥2	9.9/5.5	0.94	0.35–2.57	0.91				18.0/11.9	0.91	0.30–2.74	0.87			
Confirmed response of previous ICI treatment, PR/SD, PD	15.1/3.6	0.36	0.14–0.90	0.028	0.35	0.13–0.97	0.044	NR/10.5	0.25	0.08–0.75	0.014	0.21	0.06–0.71	0.013

Abbreviations: CI, confidence interval; ECOG, Eastern Cooperative Oncology Group; HR, hazard ratio; ICI, immune‐checkpoint inhibitor; NR, not reached; OS, overall survival; PD, progressive disease; PD‐L1, programmed death ligand 1; PFS, progression‐free survival; PR, partial response; PS, performance status; SD, stable disease; sq, squamous cell carcinoma.

**FIGURE 6:**
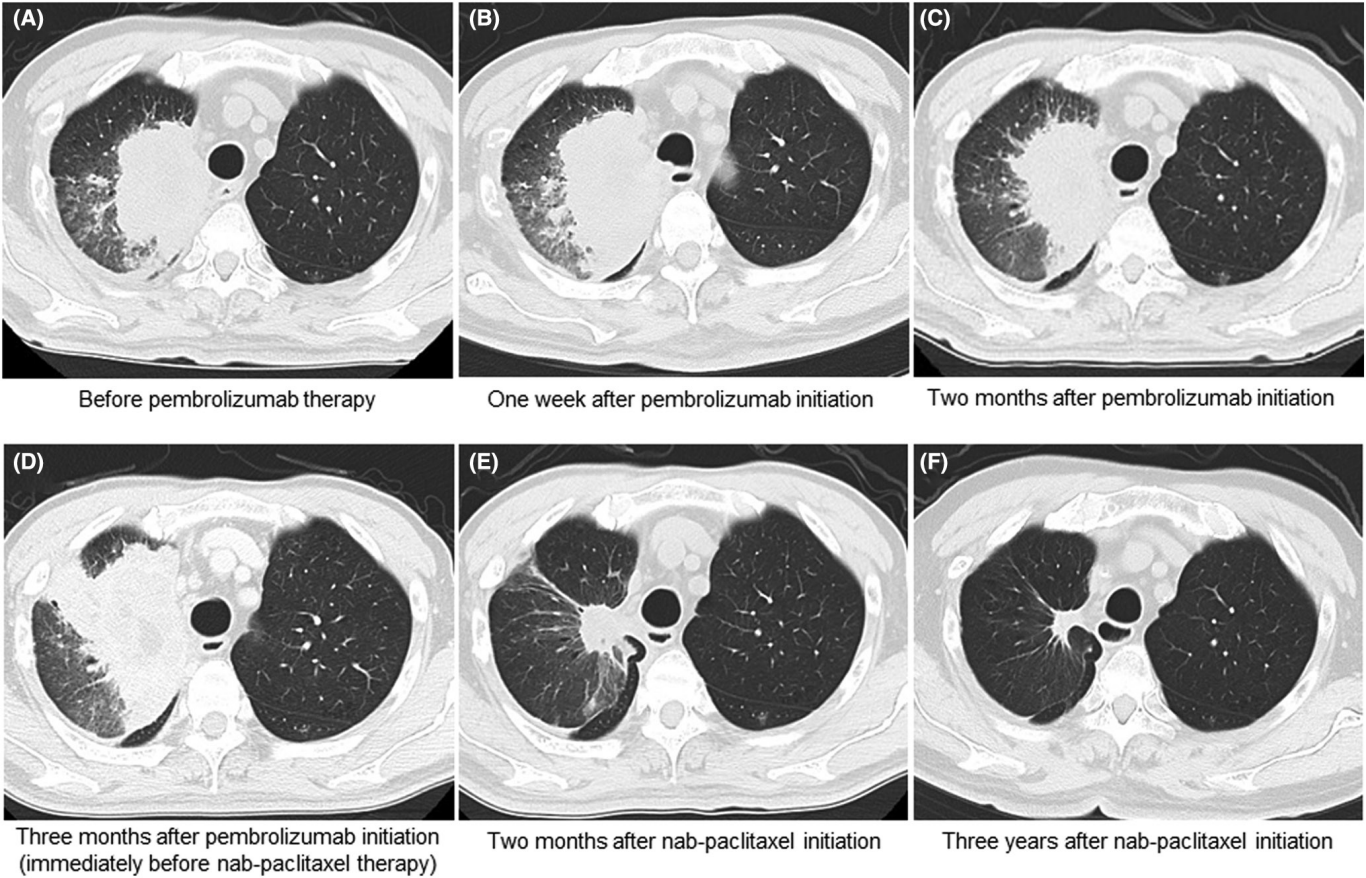
Representative CT images of a patient who showed long‐term response to nab‐paclitaxel after failure of pembrolizumab therapy. A 69‐year‐old man with an adenocarcinoma in his right upper lung lobe (A) received pembrolizumab monotherapy as a second‐line treatment. After 1 week of pembrolizumab therapy, a transient mild enlargement was observed (B), followed by a slight shrinkage 2 months later (C). However, disease progression was observed 3 months after the initiation of pembrolizumab therapy (D). He received nab‐paclitaxel immediately after pembrolizumab failure. Two months after nab‐paclitaxel monotherapy, considerable tumor shrinkage was observed (E). This response persisted until 3 years (F), with minimal adverse events, and the patients are still receiving nab‐paclitaxel therapy.

Figure 6 shows a representative case of long‐term response to nab‐paclitaxel after failure of pembrolizumab therapy.

### 3.3 Delivered chemotherapy

The median number of treatment cycles was 6 (range: 1–38). One‐stage dose reduction occurred in four patients, and two‐stage dose reduction occurred in four. Two‐stage dose reductions were due to a decline in neutrophil count and peripheral sensory neuropathy. Four patients could not continue treatment because of adverse events, and two could not continue because of patient refusal.

### 3.4 Safety

Table 5 shows hematologic and nonhematologic toxicity profiles of all 29 patients. Grade 3 or higher hematologic toxicity included leukopenia (27.6%) and neutropenia (31.0%); however, no patients with febrile neutropenia were observed. Nonhematologic toxicities observed in more than 40% of patients were peripheral sensory neuropathy (41.4%) and alopecia (58.6%), whereas Grade 3 or higher adverse events were 6.9% and 0.0%, respectively. Interstitial lung disease (ILD) was observed in three patients (10.3%), of which one (3.4%) had Grade 3. There were no treatment‐related deaths.

**TABLE 5:** Treatment‐related adverse events.

Toxicity	Any grade	Grade 3	Grade 4	Grade ≥3
Hematologic				
Leukocytopenia	15 (51.7)	6 (20.7)	2 (6.9)	8 (27.6)
Neutropenia	16 (55.2)	4 (13.8)	5 (17.2)	9 (31.0)
Thrombocytopenia	2 (6.9)	0	0	0
Anemia	16 (55.2)	0	0	0
Febrile neutropenia	0	0	0	0
Nonhematologic				
AST increase	4 (13.8)	1 (3.4)	0	1 (3.4)
ALT increase	4 (13.8)	1 (3.4)	0	1 (3.4)
Nausea	4 (13.8)	0	0	0
Anorexia	6 (20.7)	0	0	0
Joint pain	2 (6.9)	0	0	0
Myalgia	0	0	0	0
Peripheral sensory neuropathy	12 (41.4)	2 (6.9)	0	2 (6.9)
Alopecia	17 (58.6)	0	0	0
Rash	2 (6.9)	0	0	0
Interstitial lung disease	3 (10.3)	1 (3.4)	0	1 (3.4)
Fatigue	1 (3.4)	0	0	0
Paronychia	1 (3.4)	0	0	0
Creatinine increase	1 (3.4)	0	0	0
Tears	1 (3.4)	0	0	0
Eye disorders (macular edema)	1 (3.4)	0	0	0
Diarrhea	1 (3.4)	0	0	0
Bronchitis	1 (3.4)	0	0	0

*Note*: All values are given as *n* (%).

Abbreviations: ALT, alanine aminotransferase; AST, aspartate aminotransferase.

## 4 DISCUSSION

This prospective Phase 2 study evaluated the efficacy and safety of nab‐paclitaxel therapy in patients with advanced NSCLC treated with ICI alone or in combination with chemotherapy and ICI as a pretreatment and demonstrated an ORR that was higher than expected, with acceptable safety. The ORR in this study (55.2%) was higher compared to the ORR (28.1%) that was previously reported for nab‐paclitaxel as a second‐line therapy for patients with NSCLC who had not received ICI as prior therapy.^19^ Similarly, the PFS in this study tended to be better than that of our previous report (3.9 vs. 5.6 months).^19^ Furthermore, a PFS of longer than 2 years was achieved in 13.3% of patients. To the best of our knowledge, this is the first prospective study that demonstrated enhanced efficacy of chemotherapy immediately after ICI treatment failure.

Several retrospective studies have reported improved efficacy of chemotherapy after immunotherapy for NSCLC.^7^, ^8^, ^10^, ^11^, ^20^, ^21^ Because these reports included various background factors and various anticancer agents were used, the ORRs varied widely, ranging from 18% to 60%. The ORR in the current study was 55.2%, which is one of the better results among those of these studies. One reason for this is that the current study was the only prospective study; thus, more patients with preserved PS and organ function might be enrolled than those in previous studies. In addition, because there have been no previous reports of treatment with nab‐paclitaxel, nab‐paclitaxel may be more effective than other agents for the setting immediately after immunotherapy. A Phase 3 study comparing the efficacy of nab‐paclitaxel and docetaxel in previously treated advanced NSCLC proved the efficacy of nab‐paclitaxel and reported that nab‐paclitaxel was associated with a longer OS (HR 0.43) than docetaxel in patients who had received prior immunotherapy,^13^ supporting our hypothesis. The REVEL trial reported that the combination of docetaxel and ramucirumab as a second‐line therapy improved survival in patients with advanced NSCLC. Two retrospective trials of docetaxel and ramucirumab immediately after immunotherapy were reported,^8^, ^10^ with ORRs of 32.5% and 60%, respectively. These results are comparable to those of nab‐paclitaxel in the current study. However, in the REVEL study, 49% patients had Grade 3 or higher neutropenia and 16% had febrile neutropenia, compared to 20.7% and 0%, respectively, in the current study. Considering these findings, the choice of nab‐paclitaxel as a second‐line therapy after immunotherapy seems reasonable. A previous report showed a large difference in response rates to single‐agent chemotherapy immediately after immunotherapy between second‐ and third‐line therapy (46.9% vs. 25.0%).^21^ In our study, although the ORR was higher for second‐line therapy than for use of third‐line or later therapy, the ORR (50.0%) was still better for third‐line or later therapy. Therefore, among chemotherapeutic agents, nab‐paclitaxel following immunotherapy may be more effective even for late‐line therapy.

In the present study, the long‐term response of 2 years or longer was observed in 13.3% patients. Long‐term responses are generally not observed with single‐agent anticancer therapy and are often observed in the course of immunotherapy. Furthermore, long‐term responses may be a corollary of the continued efficacy of immunotherapy. In fact, a single dose of PD‐1 antibody, binding peripheral blood T cells, can persist for approximately 5 months and may continue to be effective during the next treatment.^22^ In the subset analysis of the current study, despite the fact that nab‐paclitaxel therapy was initiated after confirmation of disease progression of PD‐(L)1 inhibitor therapy in most cases, responder of previous PD‐(L)1 inhibitor treatment was associated with longer PFS and OS after initiation of nab‐paclitaxel. A recent retrospective study that evaluated the difference in efficacy of single‐agent chemotherapy with or without prior PD‐1 inhibitor therapy using propensity score‐weighted analysis found that the therapeutic effect of previous PD‐1 inhibitors was not related to the duration of response or survival to subsequent chemotherapy,^11^ which is not consistent with our present results. The possible reason for this difference is that the previous report did not include nab‐paclitaxel therapy in the analysis,^11^ and thus, the effect of prolonged PFS and OS may be specific to nab‐paclitaxel. Several explanations for the synergistic effects of paclitaxel and immunotherapy have been discussed. Paclitaxel has been reported to cause activation of dendritic cells via toll like receptors in vitro^23^, ^24^; in vivo, paclitaxel increases PD‐L1 expression level and the number of CD8^+^ T cells in tumors^25^; and in a clinical study of breast cancer, paclitaxel has been shown to increase tumor‐infiltrating lymphocytes.^26^ These findings suggest that paclitaxel may have a positive effect on the tumor microenvironment and may enhance the efficacy of immunotherapy.

Pretreatment with ICI might exacerbate the adverse events of the subsequent chemotherapy, and it was vital to evaluate the safety of treatment. Moreover, previous reports on single‐agent chemotherapy with nab‐paclitaxel for patients with NSCLC have reported a median number of treatment cycles of 3–5,^19^, ^27^, ^28^, ^29^ whereas the number of treatment cycles in this study was higher (median 6 cycles, range 1–38). This was expected to result in an increase in adverse events. However, the frequencies of peripheral sensory neuropathy and Grade 3 or higher leukopenia and neutropenia in this study were comparable to those previously reported for nab‐paclitaxel monotherapy without prior ICI.^13^, ^19^, ^27^ Despite the increased duration of nab‐paclitaxel treatment due to the longer duration of response, there was no corresponding increase in adverse events, suggesting that nab‐paclitaxel therapy immediately after ICI is well tolerated in second‐line and beyond therapies. Previous reports have reported the incidence of ILD as an adverse event with nab‐paclitaxel in the range of 4.9%–9.4%,^13^, ^19^, ^27^ and that in this study tended to be slightly higher at 10.3%. Because there is a case report of pseudoprogression and ILD in a patient treated with paclitaxel and S‐1 after completion of nivolumab therapy,^30^ ICI may increase the frequency of ILD with subsequent chemotherapy. More case series must be studied to determine whether pretreatment with ICI affects the frequency of ILD.

Although these results were clinically relevant, the study has several research limitations. First, the sample size was relatively small, and caution must be exercised regarding survival probability and frequency of adverse events. A Phase 3 randomized trial is needed to accurately validate the results of this study. However, since designing such a trial is difficult, it is necessary to validate the results in a Phase 2 trial such as the present study or in a retrospective trial with a large number of cases. Second, nab‐paclitaxel was used in this study, but further studies are needed to determine whether other agents can provide similar efficacy. Third, because only ICI monotherapy was approved in Japan during most of the study period, chemoimmunotherapy was administered as pretreatment in only a few cases in the current study. Therefore, whether nab‐paclitaxel monotherapy after chemoimmunotherapy is as effective as the present results indicate needs to be verified in the future.

In conclusion, this is the first prospective study to evaluate the effect of nab‐paclitaxel monotherapy after ICI treatment failure in patients with advanced NSCLC. The ORR of nab‐paclitaxel was clearly better than those of standard single‐agent chemotherapies, and a durable response lasting more than 2 years was observed in 13% of all cases. These results may demonstrate that ICI treatment enhances the effect of subsequent chemotherapy. The adverse events of nab‐paclitaxel immediately after ICI treatment are similar to those reported for nab‐paclitaxel therapy; however, the possible increase in the incidence of drug‐induced pneumonia should be considered cautiously. Although further studies are required, we suggest that nab‐paclitaxel after ICI is an appropriate treatment for advanced NSCLC.

## AUTHOR CONTRIBUTIONS

**Tomoaki Sonoda:** Data curation (lead); formal analysis (equal); project administration (lead); resources (equal); writing—original draft (lead). **Yukihiro Umeda:** Conceptualization (lead); funding acquisition (lead); methodology (lead); resources (equal); writing— original draft (lead). **Yoshiki Demura:** Conceptualization (equal); methodology (lead); resources (equal); supervision (equal); writing—review and editing (lead). **Toshihiko Tada:** Formal analysis (equal); resources (equal); writing—review and editing (equal). **Koki Nakashima:** Formal analysis (equal); resources (equal); writing—review and editing (equal). **Masaki Anzai:** Resources (equal); validation (lead);writing —review and editing (equal). **Makiko Yamaguchi:** Resources (equal); validation (equal); writing—review and editing (equal). **Akikazu Shimada:** Investigation (equal); resources (equal); writing—review and editing (equal). **Masahiro Ohi:** Investigation (equal); resources (equal); writing—review and editing (equal). **Chisato Honjo:** Resources (equal); software (equal); writing—review and editing (equal). **Yuko Waseda:** Resources (equal); software (equal); writing—review and editing (equal). **Masaya Akai:** Conceptualization (equal); methodology (supporting); supervision (equal); writing—review and editing (equal). **Tamotsu Ishizuka:** Conceptualization (equal); methodology (equal); supervision (lead); writing—review and editing (lead).

## CONFLICT OF INTEREST STATEMENT

The authors have no conflict of interest to declare.

## ETHICS STATEMENT

The study was approved by the medical ethics committees at each site. All patients provided written informed consent.

## Data Availability

The data that support the findings of this study are available from the corresponding author upon reasonable request.
